# Be aware of Drinkaware

**DOI:** 10.1111/add.12356

**Published:** 2013-10-28

**Authors:** Jim McCambridge, Kypros Kypri, Peter Miller, Ben Hawkins, Gerard Hastings

**Affiliations:** Faculty of Public Health and Policy, London School of Hygiene and Tropical MedicineLondon, UK1; Centre for Clinical Epidemiology and Biostatistics, School of Medicine and Public Health, University of NewcastleCallaghan, New South Wales, Australia2; School of Psychology, Deakin UniversityGeelong, Victoria, Australia3; Institute for Social Marketing, University of StirlingStirling, UK4; Institute for Social Marketing, The Open UniversityMilton Keynes, UK5

**Keywords:** Alcohol industry, corporate, policy, UK

## Abstract

In 2006, Drinkaware was established as a charity in the United Kingdom following a memorandum of understanding between the Portman Group and various UK government agencies. This debate piece briefly reviews the international literature on industry social aspects organizations, examines the nature of Drinkaware's activities and considers how the public health community should respond. Although the British addiction field and the wider public health community have distanced themselves from the Portman Group, they have not done so from Drinkaware, even though Drinkaware was devised by the Portman Group to serve industry interests. Both long-standing and more recent developments indicate very high levels of industry influence on British alcohol policy, and Drinkaware provides one mechanism of influence. We suggest that working with, and for, industry bodies such as Drinkaware helps disguise fundamental conflicts of interest and serves only to legitimize corporate efforts to promote partnership as a means of averting evidence-based alcohol policies. We invite vigorous debate on these internationally significant issues and propose that similar industry bodies should be carefully studied in other countries.

Drinkaware began as a website set up in 2004 by the Portman Group, an alcohol producer-funded organization which has attempted to influence the evidential content of policy debates through a range of tactics, including attempts to pay academics to write anonymous critiques of World Health Organization (WHO)-sponsored evidence reviews [1–3]. The Portman Group featured prominently in the previous UK government's 2004 strategy for reducing alcohol-related harms, being responsible for the provision of information on alcohol to the public [4]. This was strongly criticized at the time [5,6]. In 2006, Drinkaware was established as a separate charity ‘with the objective of positively changing public behaviour and the national drinking culture to help reduce alcohol misuse and minimise alcohol-related harm’ following a memorandum of understanding between the Portman Group, the Department of Health, the Home Office and the devolved administrations for Scotland, Wales and Northern Ireland [7].

Globalization has concentrated alcohol production among a small number of large multi-national companies. The alcohol market was worth US$979 billion in 2007, 40% of which is controlled by just 10 producers [8]. Large corporations invest heavily in a range of activities to foster national and international policy environments which favour their interests [9]. Access to internal tobacco industry documents resulting from US litigation, including those concerning the jointly owned Miller Brewing Company and Phillip Morris [10,11], shows that companies in both industries use corporate social responsibility (CSR) activities to hone their reputations, which in turn helps them to access and influence policy makers [12].

## SOCIAL ASPECTS/PUBLIC RELATIONS ORGANIZATIONS (SAPROS)

Central to the alcohol industry's CSR activities are social aspects/public relations organizations (SAPROs), set up ‘to manage issues that may be detrimental to its interests, particularly in areas that overlap with public health’ [2]. SAPROs divert attention away from population-level strategies that limit the availability, price and promotion of alcohol, and thus threaten corporate profits, towards those focused on individual responsibility [13]. SAPROs operate in policy and research by disseminating consensus statements and codes of practice [14]. They have grown very rapidly over the last decade, and Drinkaware and the Portman Group are among more than 40 alcohol-related SAPROs now operating in at least 27 countries [1]. There has not been a systematic review of SAPRO activity [15], so we draw upon experience with the Australian SAPRO, Drinkwise, to compare its *modus operandum* with that of Drinkaware.

Drinkwise was established in 2005 by the alcohol industry and funded later by the federal government of Australia in 2006. It describes itself as ‘an independent, not-for-profit organisation focused on promoting change towards a healthier and safer drinking culture in Australia’. When given public funding, critics argued that it should advocate evidence-based public health policies [16]. Instead, Drinkwise lobbied the government for ineffectual information programmes (its tagline is ‘Get the Facts’) while opposing evidence-based policies not in industry interests [17]. In 2009, 57 health experts and scientists wrote to the *Medical Journal of Australia* opposing further public funding and declaring that they would not accept funding from Drinkwise [18]. Drinkwise responded by writing individually to selected signatories, including two of the present authors, suggesting that the letter was defamatory and implying possible litigation, in the manner of the tobacco industry [19].

## WHAT DOES DRINKAWARE DO?

**Figure 1:**
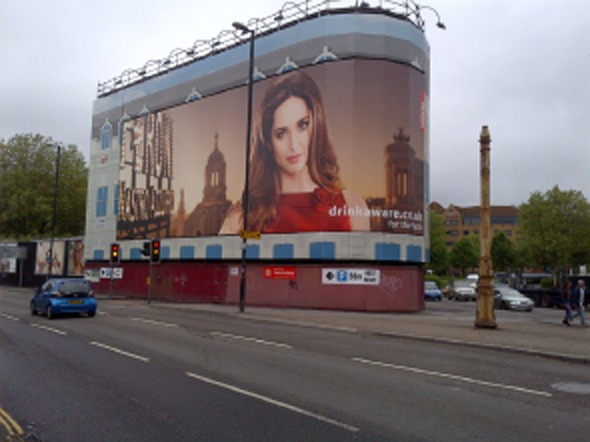
Health promotion or alcohol advertising?

Drinkaware is not publicly funded, although its activities are very similar to those of Drinkwise. It is ‘the mechanism in England for government-industry partnership on public education campaigns’ [7]. Its sophisticated multi-media website is promoted widely on alcohol packaging and marketing, although much less prominently than the core content (see Fig. 1). Drinkaware and Drinkwise have similar forms of governance, annual budgets and stated aims. Both have doctors and corporate members on their boards, and claim to provide independent, evidence-based advice to the public, particularly to help individuals make informed decisions about their drinking.

Minimum unit pricing (MUP) is a key proposal in recent British alcohol policy that was opposed strongly by sectors of the industry [20]. Aside from the commitment to MUP, the UK Government strategy placed partnership with industry at the heart of policy [21]. The result has been an energetic public debate about the evidence supporting MUP. However, the Drinkaware website, despite being promoted as the place the public should go to ‘for the facts’ [22], did not acknowledge the existence of any evidence supporting MUP. During the debates which followed the release of the government alcohol strategy it suggested that ‘Happy hours would also become slightly less cheerful’ and refers wistfully to ‘the days of the £10 crate of beer’ among other negatively toned, out-dated content—see Box 1. The website-linked tweets in Box 2 sent by Drinkaware include content that normalizes alcohol use and provides cues to drink on occasions when it may not be planned. For example, there is no British tradition of Halloween parties involving alcohol.

Box 1 Drinkaware website content*

**What's in a unit? Alcohol minimum pricing**

**Introduction**

The Chief Medical Officer recently recommended alcohol to have a minimum price per unit. How would this affect you?

Eight out of 10 people don't know the correct amount of units recommended in Government guidelines

Sir Liam Donaldson proposed that the minimum price for a unit of alcohol should be 50p per unit to curb binge drinking. For example, a 13% bottle of wine containing nine units of alcohol could not be sold for less than £4.50. Cheap supermarket promotions on bulk quantities of alcohol would also get pricier—long gone would be the days of the £10 crate of beer.

Happy hours would also become slightly less cheerful. Minimum pricing could bring an end to some drinks promotions in pubs and bars. For instance, a pint of lager with an alcohol content of 5% contains nearly 3 alcohol units, so with a minimum pricing of 50p per unit it couldn't be sold for less than £1.50.

**What exactly is a unit?**

So with all this talk about alcohol units, what exactly are they? Unfortunately it's not as simple as one drink equalling one unit. A unit is 10 mL or 8 g of pure alcohol. To put this into context, you would consume one unit of alcohol if you drank a 25 mL single measure of whisky (ABV 40%), or a third a pint of beer (ABV 5–6%) or half a standard (175 mL) glass of red wine (ABV 12%).

Many people don't have a realistic idea of how much they're drinking. In fact, Drinkaware's research shows that eight out of 10 people don't know the correct amount of units that are recommended in Government guidelines. It's recommended that men should not regularly drink more than 3–4 units a day and that women should not regularly exceed 2–3 units a day.

You can use our interactive Drinks Calculator tool to work out whether you might be drinking above the Government guidelines and also get tips on cutting down your daily intake if the results take you by surprise.

**So is minimum pricing far away?**

Whether or not the Chief Medical Officer's recommendations to put a minimum pricing of 50p on every unit of alcohol become reality remains to be seen. Gordon Brown's immediate reaction to the proposal was that putting a minimum price on alcohol would bring additional burdens on moderate drinkers.

The minimum pricing headlines ensure that the debate around the UK's alcohol culture continues to be in the spotlight. At Drinkaware, our sole aim is to provide people with the information to decide for themselves about what role alcohol plays in their lives. Whether it be helping consumers understand units or allowing them to keep track of their alcohol consumption, we will continue to provide all the facts.

**Box 2 Tweets from Drinkaware**

**Table:** 

drinkaware @**drinkaware**	12 October 2012	8.11 a.m.
It's the most popular day for work drinks! 71% of 18–24s report heading out with colleagues on a Friday. Helpful tips: http://ow.ly/eqCpe		
drinkaware @**drinkaware**	31 October 2012	7.43 a.m.
Want to make it to the witching hour and avoid feeling like a zombie tomorrow? Read our Halloween party tips… http://ow.ly/eUEV2		

Drinkaware claims that its founding memorandum of understanding [7] precludes its involvement in policy issues; yet when MUP was debated in Scotland [23], Drinkaware's written evidence to the 2009–10 Health Select Committee (HSC) alcohol enquiry argued that: ‘Behavioural change is a process which cannot happen quickly. The UK drinking culture can be changed if educational initiatives receive sufficient investment over a long enough period’ [24]. In the 2012 HSC alcohol enquiry, these claims of non-involvement in policy were repeated (e.g. ‘we are proscribed from talking about policy or lobbying’) in the face of several examples to the contrary, and incredulity among HSC members (see questions 86–109 in Ev16-18 in [25]).

## CONCERNS ABOUT DRINKAWARE

The 2012 HSC [25] noted significant concerns about industry influence on Drinkaware and the content, purposes and value of its activities—see Box 3. A long-delayed review of Drinkaware's effectiveness, which the HSC hoped would address the ‘perceived lack of independence’ from industry influence, was published early in 2013. The review, undertaken by an ‘integrated creative communications agency’ [26], was overseen by a five-member panel including Jeremy Beadles, Corporate Relations Director of Heineken UK and former Chief Executive of the Wine and Spirit Trade Association (WSTA), who oversaw a vigorous WSTA effort to dissuade the Scottish Government from introducing MUP [27,28]. The Drinkaware website also describes as ‘independent research’ an evaluation of the website by a ‘brand and communications research company’ who see their role as helping ‘our clients build stronger brands through the use of better and more relevant communications’ [29]. Both advertising agencies have histories of working with the alcohol industry.

**Box 3 2012 Parliamentary Health Select Committee views on Drinkaware [25]**

‘Chris Sorek stressed that Drinkaware is an independent charity[80], but its role is seen by some as compromised because of its links with the alcohol industry. The British Medical Association told us that

The involvement of the Drinkaware Trust in providing public health communications is a significant area of concern. This form of industry social marketing is counterproductive because industry responsibility campaigns are less effective than ones from other sources, keep messages in a commercial comfort zone, and distract attention away from more effective measures to regulate alcohol use. Industry-related messages about alcohol have been found to subtly enhance sales and company reputations. This is despite the fact that the public is cynical about the motives of corporate sponsors, and that non-governmental organisations make a more effective and credible source.’ (*Paragraph 94*) (page 32)

The HSC recommended that:

*if Drinkaware is to make a significant contribution to education and awareness over the coming years its perceived lack of independence needs to be tackled, and as part of the review that is to be held this year the Committee recommends that further steps are taken to entrench that independence. (Paragraph 97)* (page 33)

The review identifies ‘a perception of industry influence resulting in a suspicion that Drinkaware is not truly independent of the alcohol industry’ ([2.8] in [30]) and criticizes a lack of clarity about the mission and purpose of Drinkaware. It finds no evidence of ‘undue industry influence’ ([2.32] in [30]). Although recommending the building of an evidence base, it is not clear how industry actors can contribute to this when they promote information alone as sufficient for bringing about behaviour change ([9.14] in [30]). Both industry actors and the authors of the review treat funding as ‘investment’ and see the Drinkaware brand as having explicit value for the companies associated with it. For example, complaining of industry bodies that do not provide funding for Drinkaware, one funder suggested: ‘too many organisations that get to use the Drinkaware brand, get to benefit from what Drinkaware offers without actually having to put any cash up’ ([12.8] in [30]).

## WHY DOES DRINKAWARE MATTER?

Alcohol problems in the United Kingdom continue to increase while they decline in most of Europe [31]. Drinkaware has hitherto avoided concerted public health attention as an alcohol industry body. Its centrality to policy has seemed unremarkable because it merely continues in the public information role fulfilled previously by the Portman Group [4]. Despite the growing problems alcohol causes British health and society, lobbying has successfully positioned the alcohol industry very close to successive UK governments [32]. Henry Ashworth, the Chief Executive of the Portman Group, moved into that role directly from the UK Government Cabinet Office Behavioural Insights Team. Interestingly, a different situation pertains in Scotland, where the implementation of MUP is most advanced. There, the current government is not formed by a Westminster-led party and does not have close relationships with the alcohol industry [23].

It has been observed that the chapter on working with industry in the March 2012 UK Government's Alcohol Strategy ‘eschews any enhanced regulatory stance; concrete recommendations are absent … corporate friendly content on the importance of alcohol to the economy, and the need to cut red tape, i.e. deregulation’ [21] are emphasized instead. The intent of this material became apparent in late 2012 when the government launched its Consultation on the Implementation of the Strategy [33]. Impact assessments described various options for extending the availability of alcohol to places such as florists and motorway service areas. Early in 2013 it was reported that MUP would not be implemented and industry lobbying was the reason, according to a Conservative Member of Parliament with a health background [34].

## WHAT TO DO ABOUT DRINKAWARE?

Because of high levels of support from successive UK governments, Drinkaware has attracted medical and academic colleagues to support and work with it, and its materials are used in the National Health Service (NHS). In addition to examining Drinkaware's specific activities, the wider economic context needs to be considered. Both Drinkaware and the Portman Group belong to a global network of SAPROs. Corporations are legally required to protect shareholder interests, and any expenditure—including that on SAPROs or any other CSR activity—will necessarily be servant to this obligation. The alcohol industry has to find a way to reconcile pursuit of profits through increased sales and thus consumption, with the needs of governments to act to reduce the attendant increases in health and societal costs. One possible strategic direction is an aspiration to exercise ‘soft power’ in the form of ‘subtle forms of steering and control, constraining and limiting the options available for political choice’ [35]. This requires a ‘post-political’ style of partnership in which SAPROs work with governments in order to draw attention away from fundamental conflicts between economic with social and health interests [35]. SAPROs are especially useful as they can claim not to have any economic interests themselves.

We encourage NHS commissioners, public health practitioners and academic colleagues to reconsider their relationships with Drinkaware. Most would not engage with the Portman Group and we suggest that there is no obvious basis for viewing its offspring, Drinkaware, any differently. Importantly, the review of Drinkaware laments its ‘isolation within the alcohol harm reduction community’ ([2.8] in [30]), and Drinkaware is actively seeking to recruit scientists to support it (including the first author). The British public health community should bear in mind WHO's recommendation that alcohol industry bodies only be engaged in their roles as producers, distributors and retailers [36]. Key corporate tactics in influencing policy include the manufacture of doubt about unfavourable evidence [37] and creating divisions among researchers [21,38].

The evolving international literature provides new frameworks for understanding SAPRO and other CSR activities [1]. These are needed to address the historically unparalleled levels of concern about the international activities of the global alcohol industry [39,40]. SAPROs work with, and learn from, each other. The Portman Group/Drinkaware operational model, whereby a public information role is assumed by the latter SAPRO, and the former more obviously promotes industry positions on alcohol-related issues, may well be replicated in other countries.

National governments and their policy-making processes are key targets for the alcohol industry [41]. The addiction and public health research communities should examine industry influence on alcohol policies [42]. Drinkaware, like other SAPROs, appears to us to be an industry vehicle to subvert evidence-based public health policy. We propose that it is not worthy of any form of support. In the past the Portman Group divested itself of a public information function, so SAPROs may also be dispensable to the alcohol industry if they do not further its strategic objectives. We urge policy-makers to address industry influence on global and national alcohol policies [39,43,44] more assertively to reverse the mounting toll of alcohol on population health and social wellbeing.

## Notes

* This page was created on 16/03/09, was last updated on 10/05/12 and was accessed on 21/11/12. It has since been removed as part of a major redesign of the website.
